# Reforestation Under Climate Change—A Cross‐Regional Deadwood Retention Experiment to Develop Multifunctional Forests on Disturbed Norway Spruce Areas

**DOI:** 10.1002/ece3.74357

**Published:** 2026-09-16

**Authors:** Birgitta Putzenlechner, Martin Zwanzig, Claus Bässler, Markus Bernhardt‐Römermann, Janina Ebert, Simon Grieger, Philipp Koal, Henrik Oechler, Dorothea Peter, Ingolf Profft, Sascha Spaleck, Florian Steinebrunner, Alexander Tischer, Simon Thorn, Alexandra Wehnert, Xinying Zhou, Franka Huth

**Affiliations:** ^1^ Competence Centre Landscape Resilience Georg August University of Göttingen Göttingen Germany; ^2^ Chair of Forest Biometrics and Systems Analysis, Faculty of Environmental Sciences TUD Dresden University of Technology Dresden Germany; ^3^ Chair Ecology of Fungi, Faculty of Biology, Chemistry and Earth Sciences University of Bayreuth Bayreuth Germany; ^4^ Bavarian Forest National Park Grafenau Germany; ^5^ Institute of Biodiversity, Ecology and Evolution Friedrich Schiller University Jena Jena Germany; ^6^ German Centre for Integrative Biodiversity Research (iDiv) Halle‐Jena‐Leipzig Leipzig Germany; ^7^ Senckenberg Institute for Plant Form and Function (SIP) Jena Jena Germany; ^8^ Hessian Agency for Nature Conservation, Environment and Geology State Institute for the Protection of Birds Gießen Germany; ^9^ Forestry Research and Competence Centre ThüringenForst AöR Gotha Germany; ^10^ Chair of Soil Science, Faculty of Chemistry and Earth Sciences Friedrich Schiller University Jena Jena Germany; ^11^ Applied Ecology Philipps Universität Marburg Marburg Germany; ^12^ Czech Academy of Sciences Biology Centre, Institute of Entomology České Budějovice Czech Republic; ^13^ Chair of Silviculture, Faculty of Environmental Sciences TUD Dresden University of Technology Dresden Germany

**Keywords:** climate change, deadwood treatments, forest conversion, post‐disturbance management, reforestation, salvage logging

## Abstract

Extreme events such as droughts, heatwaves, storms, and the associated intensification of bark beetle outbreaks in Norway spruce forests in recent years have led to large‐scale forest loss in Central Europe. Various options for post‐disturbance management exist, from complete clearing to no intervention, but their pros and cons in the context of multifunctional forest management remain unresolved. The interdisciplinary “ResEt‐Fi” project (“Pioneering reforestation: Regional management for establishing multifunctional forests on post‐disturbed Norway spruce areas”) comprises a cross‐regional field trial to test how specific practices of deadwood retention affect the ecological conditions and subsequent reforestation of natural and artificial regeneration. It aims at generating climate‐stable mixed forests, balancing ecosystem functioning and socio‐economic benefits. Rather than presenting a conventional empirical study with finalized results, we reflect upon an integrated and transferable research framework for investigating post‐disturbance forest management across spatial scales. We present the rationale behind different deadwood management strategies, the nested experimental design of the cross‐regional field trial, the ecological response framework, upscaling approaches, and future perspectives. Based on this scientific foundation, evidence‐based recommendations and application scenarios can be developed to support forest managers and other stakeholders in selecting site‐specific post‐disturbance management strategies.

## Graphical abstract

The interdisciplinary “ResEt‐Fi” project investigates ecological and economic responses to different deadwood management practices within post‐disturbance landscapes. This article presents the rationale behind different deadwood treatments, the design of our cross‐regional field trial in Central Europe, and outlines an ecological response framework, upscaling approaches, and future perspectives that aim to provide transferable insights for post‐disturbance forest management and to support evidence‐based decision‐making for stakeholders.

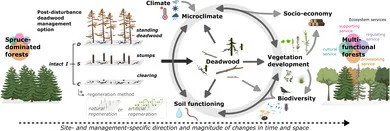

## 1 Introduction

In recent years, forests across Central Europe have been increasingly affected by climate‐induced disturbances (Forzieri et al. 2021; Patacca et al. 2023; Knutzen et al. 2025). The combination of prolonged droughts, heat extremes, and subsequent bark beetle outbreaks has triggered widespread forest disturbances and dieback, highlighting the vulnerability of current forests to compounding stressors (Senf et al. 2020; Senf and Seidl 2021; Hartmann et al. 2025). Although tree species previously considered well‐adapted to the Central European climate, such as European beech ( *Fagus sylvatica* L.), are affected as well (Leuschner 2020; West et al. 2022), particularly severe and large‐scale impacts have been observed for Norway spruce ( *Picea abies* (L.) H. Karst.) (Hlásny et al. 2021; Obladen et al. 2021). This high vulnerability is largely attributed to the widespread establishment of Norway spruce beyond its natural distribution and to historical forest management, which, driven by the objective of maximizing timber production, promoted even‐aged, mono‐layered stands with low proportions of mixed tree species through reforestation following past disturbances, reparation cuts, and silvicultural practices such as thinning from below (Spiecker et al. 2004; Nyland 2016). Although the vulnerability of Norway spruce to windthrow and increasingly frequent droughts under a warming climate has been emphasized in numerous studies (Lévesque et al. 2013; Buras and Menzel 2019; Kunert et al. 2022; Anders et al. 2025), these factors have substantially increased the predisposition of many stands to abiotic and biotic disturbances, culminating in the unprecedented disturbance intensities observed in recent years (Hartmann et al. 2025).

The high temporal dynamics of disturbances have forced forest management to act swiftly, often in an evidence‐limited space where ecological and socio‐economic outcomes remain uncertain. Post‐disturbance management strategies typically favor either salvage logging or full preservation of unlogged forest stands, while structured intermediate approaches with temporal and spatial coordination of deadwood retention remain largely absent. Instead, the commercial value of deadwood encourages salvage logging, often resulting in the near‐total removal of structural elements derived from deadwood (Keenan and Kimmins 1993). The resulting large‐scale clearings have the potential to fundamentally alter ecosystem functions (Chianucci et al. 2024). Specifically, such interventions may lead to the loss of microclimatic buffering, declines in habitat heterogeneity and biodiversity, soil degradation, or impaired ecosystem resilience over the long term (Thorn et al. 2018; Leverkus et al. 2021). However, to meet future demands for multifunctional forests under changing climatic conditions, the development of stable, resilient, and vital forest ecosystems on disturbed areas is urgently needed.

To address this situation, we initiated the collaborative research project “ResEt‐Fi” based on a cross‐regional field trial that examines various strategies for managing remaining deadwood in disturbed Norway spruce forests, focusing on the following research questions (RQ):

RQ 1 Which effects do different post‐disturbance management strategies have regarding forest functions?

RQ 2 Which site‐specific deadwood management strategy at the stand level provides the most versatile development option for climate‐resilient and multifunctional future forests?

RQ 3 How can these site‐specific deadwood treatments be translated into regional forest management and adaptation strategies to address climate change impacts more broadly?

**FIGURE 1:**
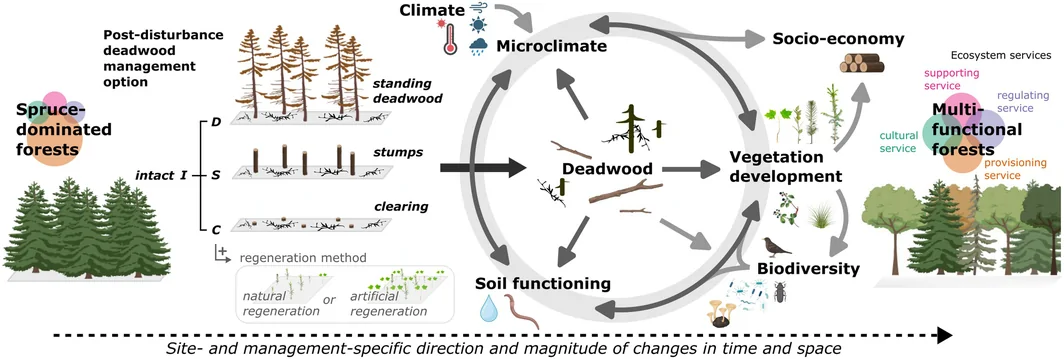
Concept of proposed feedback studied within the project “ResEt‐Fi”: Understanding how post‐disturbance deadwood management options affect interactions on disturbed areas and the paths to develop multifunctional forests in the face of climate change.

Central to this is the establishment of study areas as “real‐world forest laboratories” that simultaneously serve as experimental platforms, monitoring infrastructures, and learning environments. Such sites enable long‐term ecological observation while fostering exchange among researchers, managers, forestry training institutions, and regional stakeholders. Interdisciplinary efforts, as depicted in Figure 1, are necessary to address these questions, thereby integrating aspects such as microclimatic dynamics, soil ecology and humus development, vegetation dynamics, microbial communities and ecosystem processes, biodiversity indicators (e.g., birds and saproxylic beetles), and an economic assessment of management strategies. These questions are also intentionally framed at multiple scales, reflecting the need to bridge plot‐level ecological processes and landscape‐level management decisions. To enable spatial upscaling, multisensory remote sensing approaches are implemented, linking plot observations to landscape‐level patterns. For temporal upscaling, an individual‐based forest landscape and disturbance model is employed to explore future development trajectories under different climate and management scenarios.

Here, rather than presenting an empirical study with finalized results, our objectives are as follows: (i) to introduce the conceptual design and rationale of a joint research framework that combines ecological, silvicultural, and socio‐economic perspectives within post‐disturbance landscapes and (ii) to reflect on the perspective outcome of such integrated approaches and its potential to be scaled beyond its focal study regions. In addition, we present an analysis pipeline for our first interdisciplinary, cross‐regional assessment of effects of deadwood retention in the first 3 years after implementation of the field trial.

## 2 Methodological Framework for an Integrative Approach to Deadwood Management

### 2.1 Study Regions and Areas

**FIGURE 2:**
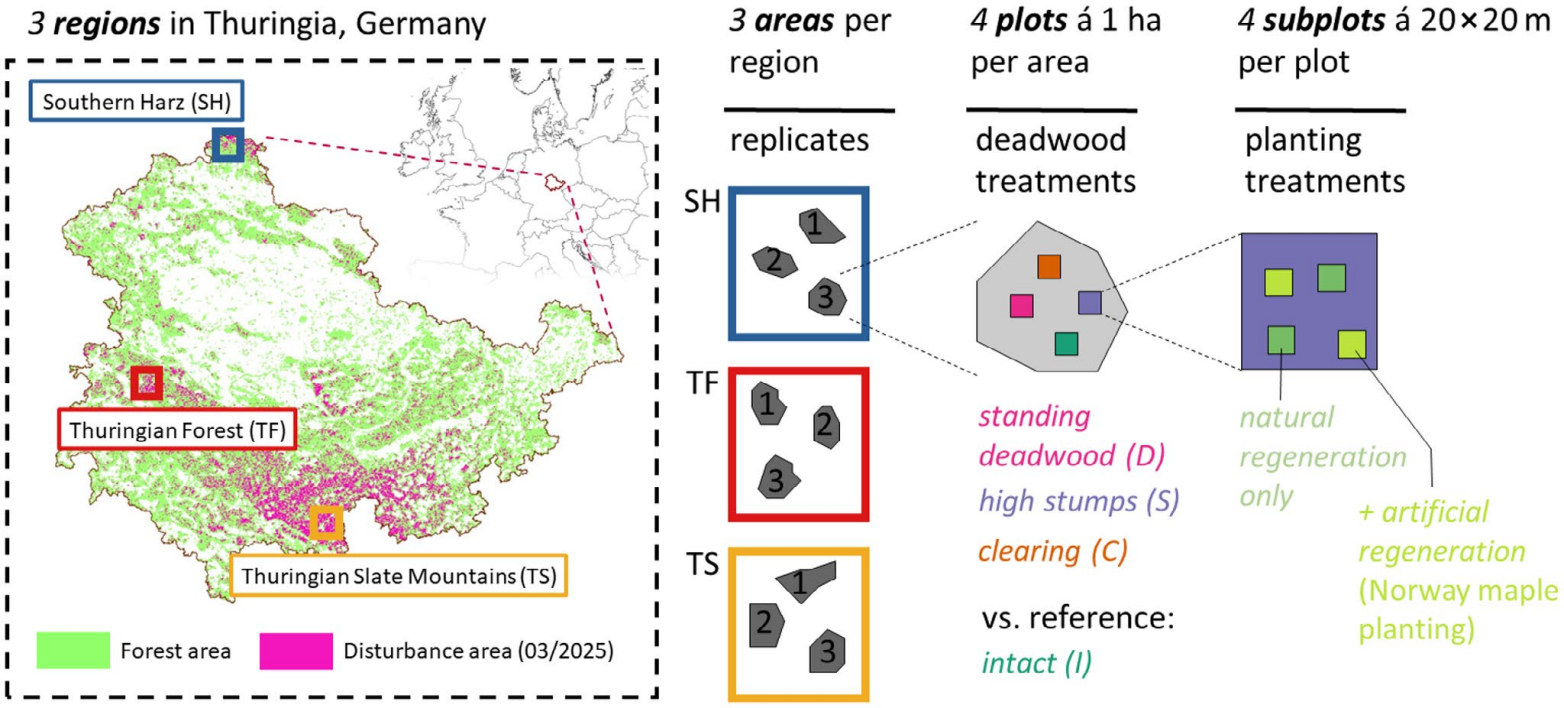
Nested experimental design with model regions within Thuringia, Germany, and areas within each region (see Table 1 for details); deadwood retention plots within each area and planting treatment subplots (2 of each category, except for intact stands) within each plot.

Research is centered in Thuringia, a federal state in Central Germany, as one of Germany's major hotspots of forest disturbance. Following the drought years beginning in 2018, extensive areas of managed Norway spruce forests were affected by large‐scale bark beetle outbreaks, providing the basis for the establishment of the cross‐regional experimental field trial. Within three model regions, a nested experimental design is applied that allows for assessing the effects of different post‐disturbance management from local to regional scale (Figure 2). The study focuses on three model regions with a high proportion of disturbed Norway spruce stands: The Western Thuringian Forest (TF), the Southern Harz Mountains (SH), and the Thuringian Slate Mountains (TS) (Figure 2). According to the 2023 forest inventory by the forest authority (“ThüringenForst AöR”), the pre‐disturbance shares of Norway spruce ranged from approximately 34% in the TF and SH regions to around 86% in TS. All three regions share a humid temperate climate, with annual precipitation sums accounting to 760 mm (SH), 880 mm (TF), and 914 mm (TS); annual mean temperatures range from 7.5°C (TS) to 8.8°C (TF) (Kronenberg et al. 2021). Cloud cover is frequent throughout the year, and snow cover may occur at higher elevations during midwinter. All three regions differ in their edaphic context (Table 1), which plays a crucial role in shaping forest regeneration dynamics.

**TABLE 1:** Overview of topographic, climatic, and geopedological characteristics of the study areas.

Region	Study area	Forest climate zone and elevation [m.a.s.l.]	Growing season length [day/year] – climatic water budget [mm/month growing season]	Parent rock (stratigraphic unit)	Soil type/humus form	Nutrient status/site moisture regime/effective soil depth	PNV
Southern Harz	SH‐1 Christianenhaus	Mid mountain zones with a humid climate; 530	110–140 – 12.5‐25	Greywackes (Devonian, Lower Carboniferous, partial Tertiary overprint)	Cambisols/Moder humus	Mesotrophic/moderate fresh/moderate deep to deep	T18 Fagus forest on acid soils
	SH‐2 Poppenberg	Mid mountain zones with a humid climate; 590	140–165 – 12.5‐25	Rhyolites and trachyandesites (Lower Rotliegend)	Cambisols and Leptosols/Moder humus	Mesotrophic to eutrophic/moderate dry to moderate fresh/shallow to moderate deep	T18 Fagus forest on acid soils; partly T171 Medio‐European neutrophile Fagus forest
	SH‐3 Rothesütte	Mid mountain zones with a humid climate; 610	110–140 – 12.5‐25	Greywackes (Devonian, Lower Carboniferous)	Cambisols/Moder humus	mesotrophic/moderate fresh/moderate deep	T18 Fagus forest on acid soils
Thuringian Forest	TF‐1 Etterwinden	Mid mountain zones with a humid climate; 630	110–140 – 12.5‐25	Mica schists, quartzites and gneisses (Cambrian)	Podzols and Cambisols/Moder and Mor humus	Dystrophic to mesotrophic/moderate fresh/moderate deep	T18 Fagus forest on acid soils
	TF‐2 Wilhelmsthal	Lower mountain zones with a humid climate; 400	140–165 – 0‐12.5	Conglomerates (Upper Rotliegend)	Cambisols/Moder humus	Mesotrophic/moderate fresh to fresh/moderate deep	T18 Fagus forest on acid soils
	TF‐3 Marksuhl	Hill terrain with a humid climate; 390	140–165 – 0‐12.5	Sandstones (Lower Buntsandstein)	Cambisols/Moder humus	Dystrophic to mesotrophic/moderate fresh/moderate deep	T18 Fagus forest on acid soils
Thuringian Slate Mountains	TS‐1 Cursdorf	Upper mountain zones with a humid climate; 700	110–140 – 12.5‐25	Mica schists, quarzites, clay and silt schists (Paleozoic)	Podzols, Cambisols and Leptosols/Moder and Mor humus	Dystrophic to mesotrophic/moderate fresh/shallow to moderate deep	T18 Fagus forest on acid soils
	TS‐2 Reichmannsdorf	Mid mountain zones with a humid climate; 660	140–165 – 0‐12.5	Mica schists, quarzites, clay and silt schists (Paleozoic)	Podzols and Cambisols/Moder and Mor humus	Dystrophic to mesotrophic/moderate fresh/moderate deep	T18 Fagus forest on acid soils
	TS‐3 Lauscha	Upper mountain zones with a very humid climate; 760	110–140 – > 25	Mica schists, quarzites, clay and silt schists (Paleozoic)	Podzols and Cambisols/Moder and Mor humus	Dystrophic to mesotrophic/moderate fresh/moderate deep	T31 Temperate mountain Picea forest

*Note:* Classification of the tree layer potential natural vegetation (PNV) follows the EUNIS classification system of the European Environment Agency (https://eunis.eea.europa.eu). Fagus refers to *Fagus sylvatica* L. Across all study areas, existing pre‐disturbance stand composition is classified as “T3N Coniferous plantation of site‐native trees.”

Post‐disturbance management strategies differ notably across the three regions: in the region “TF,” the dominant approach has been small‐scale salvage logging combined with the retention of standing dead trees. The region “SH” exhibits a mixed management history, with disturbance areas encompassing both cleared and uncleared stands. In contrast, in the region “TS,” the predominant post‐disturbance strategy has widely been the complete clearing of affected stands. This regional variability provides an important context for the experiment, while a standardized local‐scale design ensures that the ecological effects of different post‐disturbance treatments can be compared consistently across regions.

### 2.2 Experimental Design at Local Scale

**FIGURE 3:**
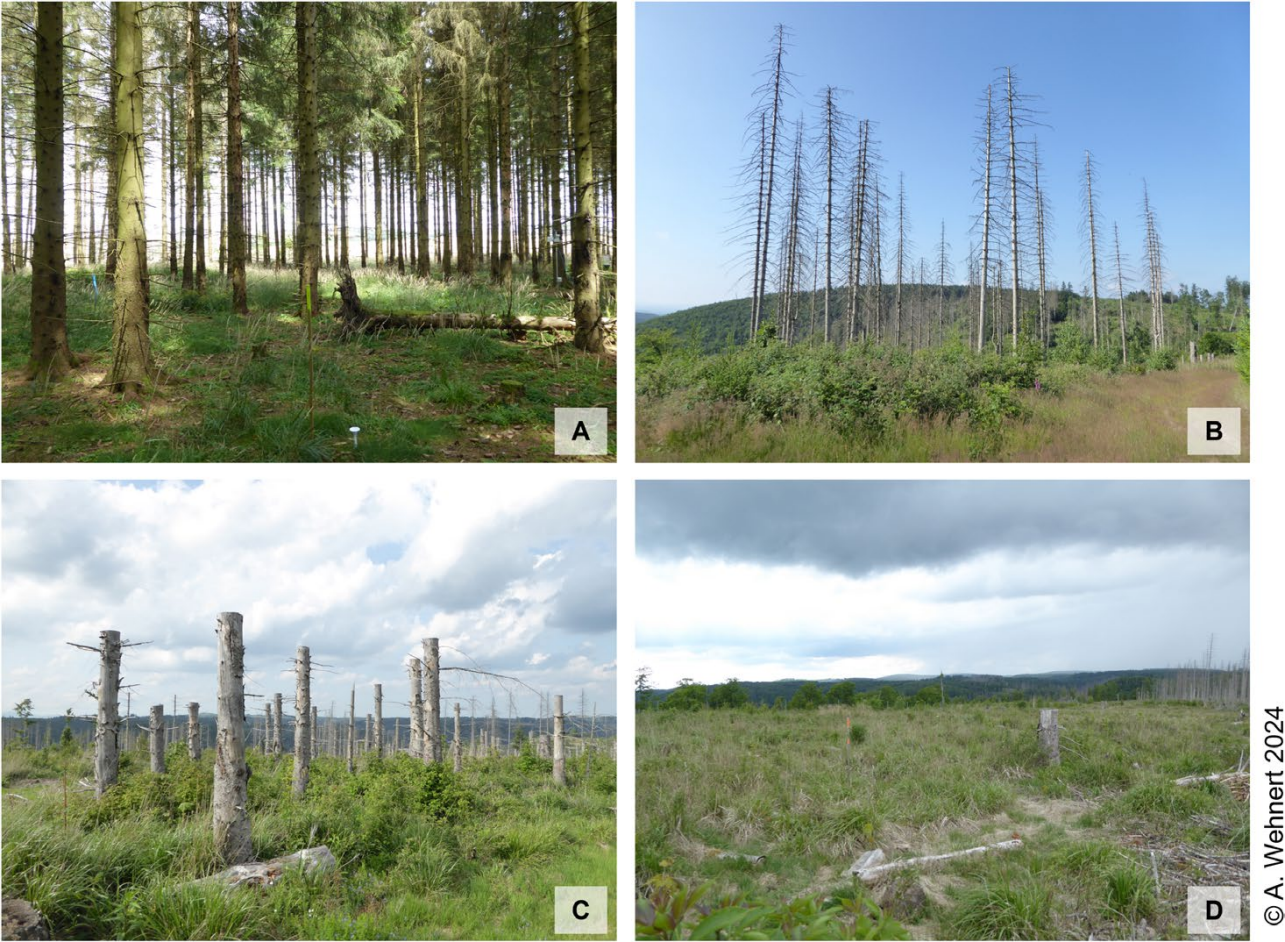
Exemplary photographs of the implemented treatment variants for the post‐disturbance deadwood retention experiment (1‐ha‐plots). (A) Undisturbed Norway spruce stand as control for intact forest “I” at study area “Rothesütte” in region SH; (B) Standing deadwood treatment “D” at study area “Etterwinden” in region TF; (C) High stumps treatment “S”; and (D) Clearing treatment “C” at study area “Christianenhaus” in region SH.

The experimental design comprises three post‐disturbance treatment variants: standing deadwood patches (“D”), high stumps (dead trees cut at 2–3 m height, “S”), and cleared areas (clearing, “C”), as well as intact Norway spruce stands (“I”). Their nested experimental approach documents individual characteristics of study areas and their variability across a total of 36 1‐ha plots (Figure 2). Silvicultural strategies for managing disturbed areas with the treatment variants D, S, and C can be compared with undisturbed Norway spruce stands as a control (I) (Figure 3). As working in areas of standing dead trees poses a significant health risk, treatment D was implemented in the form of a checkerboard pattern of areas with dead trees and areas with removed dead trees (Figure 3B). Standing deadwood patches maintain larger areas free from soil compaction and mechanical disturbance, thereby providing refugia that support soil integrity and the occurrence of sensitive taxa such as fungi. Measurements are taken on subplots that are partly within and outside of standing dead trees, which makes them accessible for maintenance and monitoring while, e.g., the microclimate is still being affected by the shelter of the standing deadwood. For treatment S, dead trees were cut at approx. 2 m, leaving standing stumps. To implement the treatment option C, all dead tree trunks were roughly cut off at their root collars and coarse woody debris (CWD) was largely removed, which left root stumps and trunk remnants with total heights of approx. 20–30 cm. In all treatments (D, S, C), fine woody debris (FWD) was left along the skid trails. As disturbance dynamics differed among study areas, the treatment variants were implemented at different times, which were documented as an important variable for subsequent analyses.

Within each 1‐ha‐plot, two (I) or four (D, S, C) subplots of 20 m × 20 m each were implemented (Figure 2), including two subplots (one for variant “I”) with natural regeneration (deadwood management without further intervention) and two subplots (one for variant “I”) with artificial regeneration (planting of Norway maple ( *Acer platanoides* L.)), where nursery‐grown seedlings were planted at a spacing of 1 × 2 m. Compared to other tree species, also within the same genus, Norway maple is rarely applied in forestry practice. In contrast, Sycamore maple ( *Acer pseudoplatanus* L.) is a tree species of great ecological importance that is widespread in the low mountain regions (Nowak and Rowntree 1990; Caudullo and de Rigo 2016). This is also reflected in the occurrence of seed trees and natural regeneration, but due to the increased occurrence of dry years and higher temperatures, Sycamore maples in particular are showing increased losses in vitality, which are further exacerbated by fungal infections (Burgdorf and Straßer 2020). Because of its lower economic importance, Norway maple is less widespread and less is known about its robustness in the regeneration phase (Carón et al. 2015). The ecological importance of Norway maple is considered to be high, especially on disturbed areas, which is due to the rapid juvenile growth, the favorable C:N ratio of the litter and the intensive rooting (Paquette et al. 2012; Roloff and Pietzarka 2014). To test the establishment and growth potential within the model regions, Norway maple was artificially planted on half the respective subplots. The absence of seed trees for Norway maple also ensures that there is no mixing with the natural regeneration in the study areas. As a thermophilic pioneer tree species with low moisture requirements, this species is characterized by high drought tolerance and a broad physiological amplitude with regard to the pH values in the topsoil (Fuchs 2021). Therefore, Norway maple is considered a suitable alternative to other pioneer tree species (e.g., *Sorbus aucuparia* L., *Salix caprea* L., *Populus tremula* L., *Betula pendula* Roth) for a rapid reforestation of disturbed areas. The subplots were not fenced to capture realistic browsing pressure, which represents a major limiting factor for natural regeneration in many Central European forests. This choice also aligns with our aim to co‐evaluate management strategies under conditions representative of actual management contexts with foresters.

### 2.3 Micro‐ and Macroclimatic Monitoring

The experimental plots and subplots are investigated through interdisciplinary monitoring to comprehensively assess effects of treatments on climate, soil, water, flora, fauna, and fungi. To ensure comparability across sites and disciplines and to capture interactions between local processes and regional dynamics, a continuous climatic data basis is fundamental for the characterization and evaluation of different management strategies.

To account for the influence of different vegetation layers, each 1‐ha plot in our study was equipped with a meteorological station to continuously record climatic conditions at plot‐scale. The meteorological stations (model: μMETOS NB‐IoT, Pessl Instruments) continuously (15‐min intervals) collect the following environmental variables on 36 research plots across the three model regions: global radiation, wind speed and direction (at 2 m height), precipitation, as well as air temperature and humidity (at 0.5 and 2 m height). These measurements fall in between classical definitions: although they include variables typically considered macroclimatic, they are taken within forest stands where canopy height and openness substantially modify radiation, wind, and temperature regimes. Thus, they represent a plot‐level, stand‐modified macroclimate.

To record microclimatic conditions and spatial variability within the 1‐ha plot, each subplot was additionally equipped with three soil moisture–temperature loggers (model: TMS4 Standard, TOMST). These data loggers measure at 15‐min intervals: soil temperature and moisture at −8 cm depth, as well as surface temperatures slightly above the ground (+2 cm) and at 15 cm above ground (+15 cm) (Wild et al. 2019).

### 2.4 Ecological Response Framework and Assessment

#### 2.4.1 Vegetation Dynamics and Forest Regeneration Potential

Forest regeneration and succession are key ecological processes determining ecosystem recovery following large‐scale disturbances. Regeneration dynamics are expected to differ among treatment variants because of differences in seed availability, regeneration microsites, vegetation development, and microclimatic conditions. Following large‐scale bark beetle outbreaks, forest regeneration is therefore largely determined by the availability of seed sources, the size of disturbed areas, and local site conditions (Wohlgemuth et al. 2019). Norway spruce often remains the predominant seed source in surrounding stands, while regeneration typically develops as heterogeneous patches depending on pre‐regeneration, microsite conditions, accompanying vegetation, and the distance to the nearest seed trees.

The mean dispersal distance of Norway spruce seeds ranges between 35 and 350 m (Piotti et al. 2009; Gratzer and Waagepetersen 2018), resulting in spatial gradients in regeneration density that are further modified by climatic and light‐related edge effects associated with remaining trees or standing deadwood. Early‐successional, light‐demanding tree genera such as birch, poplar, willow, larch, and pine rapidly colonize disturbed areas but require suitable regeneration microsites, including vegetation‐free or bare mineral soil, for successful establishment (Tiebel et al. 2018). Successful regeneration further depends on seed availability and the temporarily activated soil seed bank. Although wind‐dispersed pioneer species generally exhibit greater dispersal distances, they also form spatially heterogeneous regeneration patterns reflecting favorable regeneration microsites (Schupp 1995; Candotti et al. 2025). Where mixed tree species are successfully established, strong interspecific competition during the regeneration phase is expected (Candotti et al. 2025).

Vegetation succession is expected to play a central role in shaping regeneration trajectories. Following canopy loss, increased light availability promotes rapid vegetation development. Forest understory species may initially persist after disturbance and coexist with opportunistic or competitive species such as *Rubus* spp. and *Calamagrostis* spp. (Swanson et al. 2011). The rapid spread of these species may restrict the regeneration window for tree seedlings through competition for light, water, and nutrients. During the early stages of succession, shrubs and grasses frequently dominate before pioneer tree species gradually outgrow them (Leuschner and Ellenberg 2018). Previous studies demonstrated that post‐disturbance deadwood management influences these successional pathways. Salvage logging in bark beetle‐affected forests increased species richness and grass cover while reducing shrub cover in the short term (Fornwalt et al. 2018), whereas forest herbs persisted beneath standing dead trees, and grasses became dominant (Jonášová and Prach 2008). Together, these studies indicate that deadwood retention may influence regeneration not only by creating favorable regeneration microsites but also indirectly through its effects on vegetation succession and competitive interactions.

The regeneration of tree species is recorded spatially explicit, taking deadwood into account. The development of regenerated tree species is evaluated based on age, height growth, root collar diameter, and vitality. Ground vegetation succession is assessed through repeated vegetation surveys complemented by measurements of biophysical vegetation properties, including leaf area index (LAI) and the fraction of absorbed photosynthetically active radiation (FAPAR). By integrating these observations across contrasting deadwood treatments, the framework enables assessment of how post‐disturbance management shapes regeneration dynamics, vegetation succession, and the development of structurally diverse forest stands during the early stages of ecosystem recovery.

#### 2.4.2 Soil Ecological Processes

Large‐scale forest disturbances lead to abrupt changes in ecological site conditions. The sudden loss of canopy alters litter input, temperature, and moisture regimes, inducing shifts in soil fauna and microbial activity that affect humus dynamics and soil ecological processes (Salmon et al. 2008; Gomoryova et al. 2017; Choma et al. 2021). In Norway spruce forests, raw humus and moder forms dominate and are characterized by thick acidic litter layers, high C:N ratios, and low biological activity (Zanella et al. 2018; Buresova et al. 2021). Following disturbance, vegetation succession initiates change in litter quality and decomposition processes, promoting transitions toward moder or mull humus forms and altering nutrient cycling, soil organic carbon dynamics, soil pH, and microbial communities (Bernier and Ponge 1994; Labaz et al. 2014; Choma et al. 2021; Klein‐Raufhake et al. 2025).

The extent to which deadwood is retained or removed after disturbance may further influence these trajectories. Deadwood contributes both labile and recalcitrant organic substrates, regulates microclimatic conditions, and provides habitat for saproxylic organisms, thereby affecting decomposition processes and nutrient cycling (Merganičová et al. 2012; Bani et al. 2018; Klein‐Raufhake et al. 2025). Consequently, soil ecological responses are closely linked to vegetation development, microclimate, and post‐disturbance management. Initial findings on deadwood treatment‐specific changes in soil chemistry have recently been reported by George et al. (2026), while the broader interdisciplinary monitoring will allow these responses to be linked to belowground biological processes and ecosystem functioning.

Soil ecological processes are assessed through fine‐scale humus layer sampling, humus‐form mapping, and complementary measurements of topsoil properties. By integrating these observations with vegetation surveys, microclimate measurements, and experimental treatments, the framework enables assessment of how alternative deadwood management strategies influence soil development and ecosystem functioning during forest recovery.

#### 2.4.3 Microbial Communities and Biodiversity

Microbial communities and forest biodiversity are essential for ecosystem processes and are closely linked to structural changes following bark beetle disturbances. While vegetation succession drives long‐term ecosystem recovery, deadwood provides essential habitat and resources for microorganisms and forest‐dwelling species, thereby influencing decomposition, nutrient cycling, and biodiversity. The response of microbial communities depends strongly on their ecological functions. Ectomycorrhizal (ECM) fungi, which form mutualistic associations with trees, generally decline in abundance and diversity following large‐scale disturbances due to the loss of suitable host trees (Štursová et al. 2014; Veselá et al. 2019; Kosunen et al. 2020). Their long‐term recovery is closely linked to forest regeneration and the successional development of tree communities. In contrast, saprotrophic fungi typically benefit from the increased availability of dead organic matter following disturbance, although responses differ among substrates (Štursová et al. 2014; Masch et al. 2025). Since fungal diversity generally increases with substrate quantity and heterogeneity (Baber et al. 2016; Krah et al. 2018; Uhl et al. 2022), deadwood retention is expected to promote both fungal diversity and ecosystem functioning. Bacterial communities are generally more resistant to disturbance, with previous studies reporting comparatively small changes in species richness but shifts in community composition driven by changes in soil moisture, pH, and C:N ratio (Štursová et al. 2014; Custer et al. 2020).

Specifically, altered light conditions and increasing deadwood availability create habitat for disturbance‐adapted specialist species (Thorn et al. 2016; Viljur et al. 2022). Structural legacies, particularly standing deadwood and downed woody debris, provide habitat for organisms that depend on decaying wood for nutrition, reproduction, or shelter (Thorn et al. 2016, 2020). Retaining standing deadwood may provide nesting habitat for cavity‐nesting birds and temporary refugia for ground‐ and shrub‐nesting species during forest regeneration (Castro et al. 2010). Likewise, canopy openness and deadwood availability are key drivers of arthropod diversity, particularly for saproxylic beetles (Perlík et al. 2023), while the retention of high stumps represents an important but probably overlooked habitat for wood‐dependent species (Lackner et al. 2024). In addition, the natural regeneration of shrubs and herbaceous vegetation provides important food resources and habitat for higher trophic levels.

Biota (microbial communities, birds, arthropods) and biodiversity are assessed alongside vegetation development, soil ecological processes, and structural habitat characteristics to quantify ecosystem responses to contrasting post‐disturbance management strategies. Integrating these components enables assessment of how nuances of deadwood retention influence biodiversity conservation, ecosystem functioning, and forest recovery across multiple trophic levels.

### 2.5 Analysis Pipeline

To address RQ1, this study will perform an analysis integrating core observations from the entire research consortium to resolve the joint ecosystem responses to deadwood retention treatments within the first 3 years after implementation of the field trial.

#### 2.5.1 Integrative Hypothesis

Microclimatic buffering, seedling growth, soil health, and biodiversity, except in vegetation, are positively correlated with retained deadwood and thus differ by treatment, although greatly varying by region.

#### 2.5.2 Analytical Approach

Partial distance‐based Redundancy Analysis (db‐RDA) will be performed considering an ensemble of multifaceted response variables (Table 2), constraining by deadwood treatment (I, D, S, C) and conditioning on the nested structure (region/area). This method is suited to visualize and test the effects of treatments on multivariate continuous response variables while accounting for nested structure (Legendre and Anderson 1999). Input for the db‐RDA will be a Bray–Curtis dissimilarity matrix, suitable for ecological data with non‐linear relationships and compositional structures (Legendre and Anderson 1999). It will be calculated with the R package “vegan” (Oksanen et al. 2026) using response variables standardized by z‐transformation (R function “scale”). Standardization ensures that all response variables contribute equally to the Bray–Curtis dissimilarity calculation, regardless of their original scales or units. The *Z*‐scores are robust for variables with different distributions and scales and not sensitive to outliers such as range standardization.

**TABLE 2:** Overview of response variables used to address the integrative hypothesis for research question 1.

Response variable	Sampling	Processing
Thermal exceedance hours	Automated measurements (15‐min interval) at 15 cm height above the surface (for air temperature) and up to −14 cm depth (for soil moisture), at three random points in each subplot (=12 measurements per timestep in C, D, S and 6 in I)	Mean sum of hours of +15 cm air temperature records equal or above 35°C^a^ in 2024–2026
Spring frost exposure		Mean sum of hours of +15 cm air temperature records below 0°C in April to May in 2024–2026
Cumulative soil moisture deficit		Sum of soil moisture index (SMI) values equal or below 0.5^b^ in April to September in 2024–2026 Raw data is converted to volumetric water content using the soil moisture calibration function implemented in myClim (Man et al. 2023) and site‐specific soil parameters
Humus mass of forest floor	Humus‐form mapping and sampling according to Zanella et al. (2018) considering morphological and structural characteristics of humus layers. Sampling for the material of each layer using a metal frame to achieve areal‐based mass sampling. Three sampling locations at each plot. Sampling in April and May 2025	Living plant material and stones are removed from humus layer samples. Material is dried at 40°C for 5 days. The mass of each layer is determined using an analytical balance and summed for each sampling location. Mass is divided by sampling area and averaged from three replicates for each layer
‍Topsoil C:N ratio	Soil sampling of the humus layers with fine depth resolution of morphological properties (Oi, Oe, and Oa horizons, as well as the transition horizons), and of the mineral soil according to 5 cm depth intervals, in April and May 2025, in each plot. Sampling is carried out either based on the sampling area (forest floor humus layers) or on volume	Sampling and raw data are processed by consideration of analytical standards^c^ and daily analysis factor of elemental analyzer Elemental ratios are calculated as molar ratios and averaged for topsoil
‍Shoot length increment	Measurement of the shoot length for the year 2025 for all planted Norway maple saplings in one subplot per plot (Ammer et al. 2004)	Calculation of the average shoot length per plot
Competing vegetation cover	Visual estimation of the area covered by vascular plant species in each plot. Survey conducted at the peak of the growing season in 2024. We use the method by Braun‐Blanquet (1964) with the following cover intervals: < 1%, 1%–3%, 4%–5%, 5%–10%, 10%–25%, 25%–50%, 50%–75%, and 75%–100%, as proposed by Pfadenhauer (1997)	Sum of per plot area shares covered by *Calamagrostis* spp. and *Rubus* spp., which are strong competitors for tree regeneration
Vascular plant diversity		Plot‐level alpha diversity is quantified^d^
Tree regeneration diversity	For each plot, the number of naturally regenerated plants is counted for the year 2025, differentiated by tree species	Plot‐level alpha diversity is quantified^d^
Bacterial diversity	Bacterial communities are sampled on each plot across the same substrate types as fungi between October of 2023 and October of 2025	DNA is extracted and bacterial communities characterized by amplicon sequencing of the 16S rRNA region. Sequences are processed as above and taxonomically assigned against the SILVA database. Plot‐level alpha diversity is quantified^d^
Fungal diversity	Fungal communities are sampled on each plot across multiple substrate types including soil, deadwood, litter, dung, and carrion using substrate‐specific protocols between October of 2023 and October of 2025	DNA is extracted and fungal communities are characterized by amplicon sequencing of the ITS2 region. Sequences are quality‐filtered, denoised, and taxonomically assigned against the UNITE database. Reads from all substrate samples are pooled per plot prior to diversity estimation. Plot‐level alpha diversity is quantified^d^
Arthropod diversity	Beetles are sampled by pitfall traps and flight interception traps on each plot from March/April until the beginning of September 2024 and 2025. Pit fall traps are only implemented in 2024, whereas flight interception traps are installed in 2024 and 2025	Beetles are identified to species level by Specialists and are classified as non‐saproxylic or saproxylic according to Schmidl and Bußler (2004). Traps are pooled per plot prior to diversity estimation. Plot‐level alpha diversity is quantified^d^
Avifaunal diversity	Birds are recorded by standardized point‐count surveys on each plot five times per year between March and June in 2024 and 2025	Plot‐level alpha diversity is quantified^d^

^a^ Low range of break point temperatures found for different temperate tree species (Münchinger et al. 2023).

^b^ SMI is the ratio of total storage available to plants, calculated by the given volumetric water content θ, water content at permanent wilting point *θ*_PWP_ and water content at field capacity *θ*_FC_: SMI = (*θ*–*θ*_PWP_)/(*θ*_FC_−*θ*_PWP_). For this comparison, *θ*_PWP_ and *θ*_FC_ will be calculated separately for each plot based on its specific soil texture derived from soil surveys. Following the general theoretical SMI framework (Seneviratne et al. 2010) and empirical insights from sapling experiments of Central European tree species (Niemczyk et al. 2023), we assume the conservative estimate of a SMI threshold of 0.5 to indicate soil moisture limited conditions for plant growth.

^c^ Soil sampling in the field. Transport under cooled conditions to the lab. Remove stones and living plant material (and sieving for mineral soil samples). Subsampling and drying the subsamples at 60°C for 4 days. Grinding and analysis using a Vario Max cube elemental analyzer by dry catalytic combustion (Elementar) for C and N.

^d^ As Hill q1 (effective number of species, equivalent to the exponential of Shannon entropy) at a standardized sample coverage, integrating both richness and abundance structure into a single plot‐level estimate.

We will visualize the results using a db‐RDA biplot, displaying plot scores, response variable vectors, and treatment centroids with 95% confidence ellipses. This will allow us to interpret both the strength and direction of treatment effects on the multivariate response. In addition, we will implement restricted permutation tests (999 permutations) using the R package “permute,” ensuring permutations are only performed within regions and areas to respect the nested design. We will report on the proportion of constrained inertia (variance explained by treatment), the pseudo‐*F* statistic, and the permutation‐based *p*‐value for the treatment effect.

### 2.6 Upscaling

**FIGURE 4:**
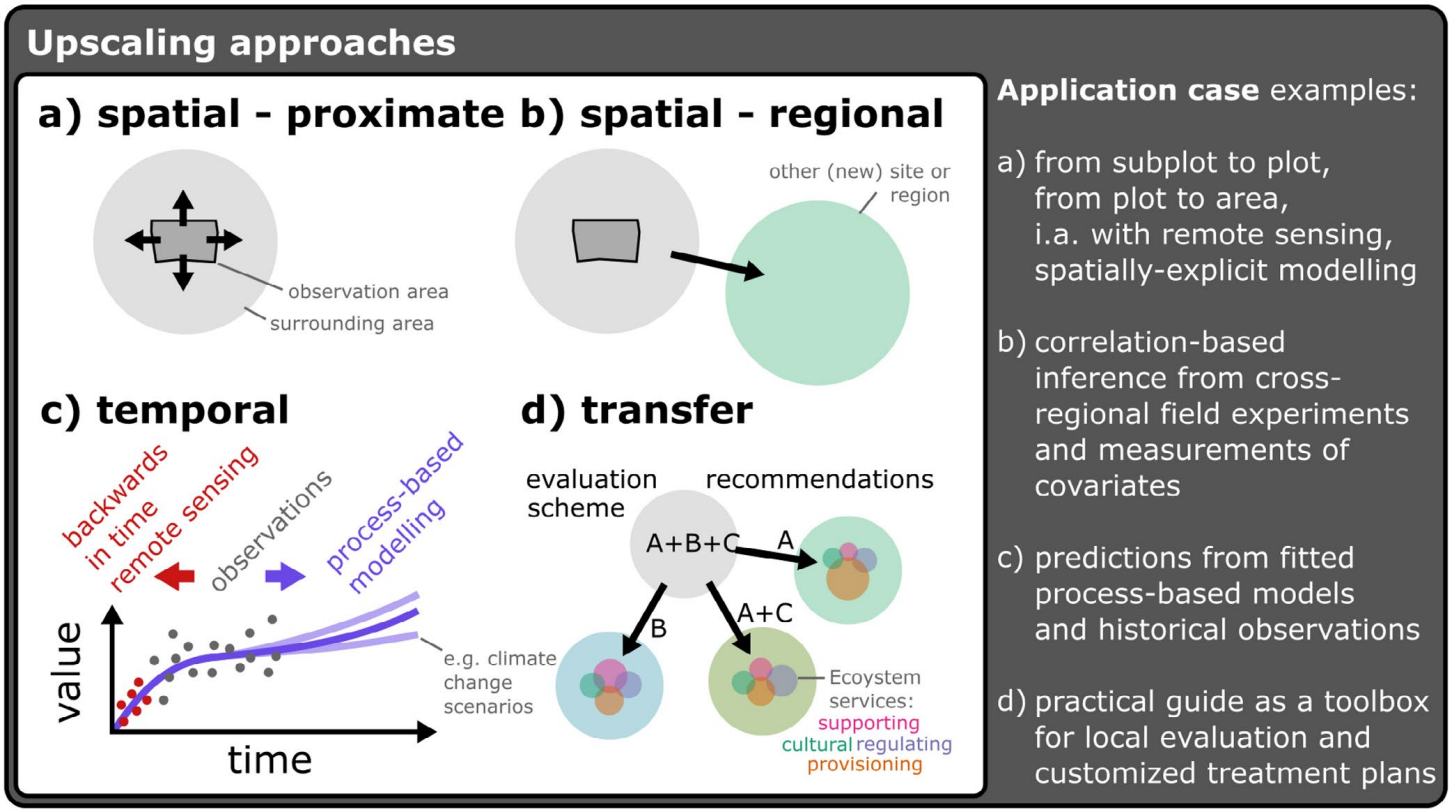
Upscaling approaches serving different dimensions and purposes, enabled by interdisciplinary research based on a cross‐regional experimental setup. Upscaling can address the transfer of observed conditions and relationships to proximate, neighboring areas (a), to other regions (b), and across time (c). It can also address the transfer of recommendations to new scenarios of practical interest for forestry (d).

While the experimental approach and analysis outlined above will provide detailed insights into ecosystem responses at the plot scale, broader application (RQ 2 and RQ 3) requires upscaling across both space and time (Figure 4). By jointly monitoring vegetation dynamics, soil ecological processes, and biodiversity, the project captures complementary ecosystem responses that can subsequently be integrated with remote sensing and agent‐based modeling for spatial and temporal upscaling. Remote sensing enables the transfer of ecological responses to the landscape scale, whereas modeling extends the experimental observations to long‐term forest development under alternative post‐disturbance management strategies and future climate scenarios.

#### 2.6.1 Spatial Upscaling With Remote Sensing

Combining UAV‐ and satellite‐based observations enables the spatial characterization of vegetation recovery and thermal conditions across larger forest landscapes, providing the basis for transferring ecological responses from the plot to the landscape scale. Satellite remote sensing provides the regional perspective on disturbance dynamics and post‐disturbance ecosystem functioning. Multi‐temporal Sentinel‐2 observations on forest disturbance dynamics can be combined with Landsat‐derived LST to characterize the spatial variability of forest disturbance and associated thermal conditions across the study regions (Grieger et al. 2025). The integration of these observations with topographic and pedological site characteristics enables the identification of environmental gradients and forest areas with an elevated risk of increased thermal stress, thereby supporting the interpretation of regeneration conditions at the landscape scale.

At the plot scale, UAV observations complement the field monitoring by resolving fine‐scale spatial variability in vegetation recovery and surface temperatures. Repeated UAV surveys provide detailed information on treatment‐specific vegetation development and microclimatic heterogeneity (Putzenlechner et al. 2025).

Building on these approaches, cross‐regional monitoring provides the basis for spatially upscaling regeneration‐relevant microclimatic conditions. By combining remotely sensed variables with the extensive network of microclimate loggers and additional structural and ecological predictors, such as deadwood availability, vegetation development, soil properties, and biodiversity indicators, predictive models can be developed to transfer plot‐scale observations to the landscape scale.

#### 2.6.2 Temporal Upscaling With Agent‐Based Modeling

Climate model projections consistently point toward warmer temperatures, drier summers, and more humid winters across the model regions (MDK, v1.0; Struve et al. 2020). This will challenge the drought resistance of both newly planted Norway maple trees and natural regeneration on our study plots, as well as neighboring stands that have been less affected so far. While the monitoring network captures the early stages of ecosystem recovery, long‐term forest development under different management strategies and future climate conditions requires a modeling approach. Agent‐based forest landscape models provide a suitable framework for integrating ecological processes observed in the field with climate projections to evaluate long‐term forest dynamics. The individual‐based forest landscape model *iLand* (Seidl et al. 2012; Rammer et al. 2024) is used for the temporal upscaling of ecosystem responses. It simulates the establishment, growth, and mortality of individual trees based on first principles, accounting for the interception of light, water uptake, allometric plasticity, and species‐specific life‐history traits resolving a tree's local response to daily changes in climatic conditions. These and further environmental conditions can be defined separately for resource units forming the simulated landscape (Rammer et al. 2024). In addition to tree population dynamics, the model represents key ecosystem processes relevant for post‐disturbance forest recovery, including regeneration, vegetation succession, and disturbance interactions. This process‐based representation allows the evaluation of alternative management strategies under changing climatic conditions.

Using such a simulation model, the empirical observations on soil properties, microclimate and regeneration, from the initial post‐disturbance status to continued observations in treatment plots, can be assessed integrally and transferred into long term predictions of forest growth, composition, structure and associated ecosystem functions such as timber production, carbon sequestration, site protection and biodiversity conservation in order to address our research questions. Simulations are performed for different climate scenarios mimicking empirically realized experimental treatments, and, in addition, for synthetic experimental conditions in order to reveal the effect of factors such as size of disturbance and seed tree availability, and to infer how they translate into necessities and opportunities of post‐disturbance management regarding the temporal and spatial aspects of planting.

## 3 Perspective

### 3.1 Opportunities and Limitations

Beyond addressing the specific research questions, the interdisciplinary framework provides three complementary perspectives. First, it enables an integrated assessment of post‐disturbance management strategies with different levels of deadwood retention across representative regions in Central Europe. Second, it provides the basis for developing evidence‐based recommendations and evaluation schemes for salvage logging intensity and the retention of different deadwood structures. Third, by combining field observations with remote sensing and modeling, it supports the upscaling of local ecological responses across spatial and temporal scales, thereby facilitating the transfer of site‐specific findings into practical forest management and climate adaptation strategies. Together, these components provide the scientific foundation for translating process‐based ecological understanding into practical decision support for climate‐resilient forest management.

The strength of this framework lies in the combination of a cross‐regional experimental design with interdisciplinary monitoring. According to the orthogonality–representativeness–comprehensiveness concept (Verheyen et al. 2017), multi‐regional experimental designs improve the ability to disentangle environmental drivers while simultaneously increasing the representativeness of observations and broadening the spectrum of ecological questions that can be addressed. Moreover, as the study sites are located in actively managed forests rather than protected areas, the project provides opportunities for continuous exchange and mutual learning between researchers, forest managers, and other stakeholders. This holds the potential to integrate ecological findings with economic assessments and management objectives to evaluate trade‐offs between biodiversity conservation, ecosystem functioning, and timber production.

At the same time, two main challenges remain. First, forest recovery spans decades, whereas the current monitoring primarily captures the initial stages of succession and forest recovery. These observations nevertheless provide an essential baseline against which future ecological change can be assessed through repeated surveys and long‐term monitoring. Second, while the present framework primarily focuses on how abiotic conditions shape ecological responses following disturbance, reciprocal biotic–abiotic feedback is expected to emerge over longer temporal scales. Capturing these dynamic interactions, together with changing climatic conditions and disturbance regimes, represents an important opportunity for future monitoring and model development.

### 3.2 Transferability Across Central Europe

The study regions comprise low mountain ranges, spanning lower to upper montane zones with humid to very humid climates and predominantly acidic, silica‐rich soils of low to moderate nutrient availability. This environmental gradient provides a basis for transferring the experimental framework and its findings to comparable forest landscapes, including large parts of the German Central Uplands, the Bohemian Massif, and the Rhenish Massif. As Norway spruce currently accounts for between 34% and 86% of the forest cover within the study regions, the project addresses forest conditions that are widespread across many disturbance‐prone areas of Central Europe.

Nevertheless, the transferability of specific ecological responses depends on local site conditions and forest composition. Factors such as climate, soil properties, seed tree availability, seed banks, and the composition of understory, microbial, and faunal communities are influenced by site history and may alter regeneration trajectories and ecosystem responses. Likewise, the present study focuses on post‐disturbance management in Norway spruce forests. Extrapolation to other forest types should therefore be made cautiously, as tree species differ in disturbance responses, wood chemistry, decomposition dynamics, and the ecological communities they support. Nevertheless, the integrated framework provides a transferable approach for investigating post‐disturbance forest recovery across comparable environmental settings and can readily be adapted to address region‐ and species‐specific management questions.

### 3.3 Future Directions

The proposed framework establishes the scientific foundation for an evidence‐based assessment of post‐disturbance forest management across environmental gradients and spatial scales. As the project progresses, the interdisciplinary observations and modeling approaches will be synthesized into a decision‐support framework that links ecological responses to management objectives under contrasting site conditions and future climate scenarios. Rather than promoting a single deadwood management strategy, this framework aims to support context‐specific decisions by considering trade‐offs among forest regeneration, biodiversity conservation, ecosystem functioning, and operational feasibility. Such a “deadwood retention toolbox” could provide forest managers with practical guidance for selecting management options tailored to local conditions and desired ecosystem services.

## Author Contributions

**Birgitta Putzenlechner:** conceptualization (equal), funding acquisition (equal), investigation (equal), resources (equal), supervision (equal), visualization (supporting), writing – original draft (lead), writing – review and editing (lead). **Martin Zwanzig:** conceptualization (equal), funding acquisition (equal), investigation (equal), resources (equal), supervision (equal), visualization (lead), writing – original draft (lead), writing – review and editing (lead). **Claus Bässler:** conceptualization (equal), funding acquisition (equal), resources (equal), supervision (equal), writing – review and editing (equal). **Markus Bernhardt‐Römermann:** conceptualization (equal), funding acquisition (equal), resources (equal), supervision (equal), writing – review and editing (equal). **Janina Ebert:** investigation (equal), writing – original draft (equal), writing – review and editing (equal). **Simon Grieger:** investigation (equal), writing – original draft (equal), writing – review and editing (equal). **Philipp Koal:** conceptualization (equal), funding acquisition (equal), visualization (supporting), writing – original draft (equal), writing – review and editing (equal). **Henrik Oechler:** investigation (equal), writing – original draft (equal), writing – review and editing (equal). **Dorothea Peter:** investigation (equal), writing – original draft (equal), writing – review and editing (equal). **Ingolf Profft:** conceptualization (equal), funding acquisition (equal), project administration (equal), resources (equal), supervision (equal), writing – review and editing (equal). **Sascha Spaleck:** investigation (equal), writing – review and editing (equal). **Florian Steinebrunner:** investigation (equal), writing – original draft (equal), writing – review and editing (equal). **Alexander Tischer:** conceptualization (equal), funding acquisition (equal), resources (equal), supervision (equal), writing – original draft (equal), writing – review and editing (equal). **Simon Thorn:** conceptualization (equal), funding acquisition (equal), resources (equal), supervision (equal), writing – review and editing (equal). **Alexandra Wehnert:** investigation (equal), visualization (supporting), writing – original draft (equal), writing – review and editing (equal). **Xinying Zhou:** investigation (equal), writing – original draft (equal), writing – review and editing (equal). **Franka Huth:** conceptualization (equal), funding acquisition (equal), investigation (equal), resources (equal), visualization (supporting), writing – original draft (equal), writing – review and editing (equal).

## Funding

This work was supported by Bundesministerium für Forschung, Technologie und Raumfahrt (BMFTR), 033L304A‐F.

## Conflicts of Interest

The authors declare no conflicts of interest.

## Data Availability

Data providing information on characteristics of the study areas for the post‐disturbance deadwood management project “ResEt‐Fi” are available through the following repository: https://zenodo.org/records/17587138.
